# What Makes Sports Fans Interactive? Identifying Factors Affecting Chat Interactions in Online Sports Viewing

**DOI:** 10.1371/journal.pone.0148377

**Published:** 2016-02-05

**Authors:** Minsam Ko, Jaeryong Yeo, Juyeong Lee, Uichin Lee, Young Jae Jang

**Affiliations:** 1 Department of Knowledge Service Engineering, KAIST, Daejeon, Republic of Korea; 2 Department of Industrial and Systems Engineering, KAIST, Daejeon, Republic of Korea; University of Verona, ITALY

## Abstract

Sports fans are able to watch games from many locations using TV services while interacting with other fans online. In this paper, we identify the factors that affect sports viewers’ online interactions. Using a large-scale dataset of more than 25 million chat messages from a popular social TV site for baseball, we extract various game-related factors, and investigate the relationships between these factors and fans’ interactions using a series of multiple regression analyses. As a result, we identify several factors that are significantly related to viewer interactions. In addition, we determine that the influence of these factors varies according to the user group; i.e., active *vs*. less active users, and loyal *vs*. non-loyal users.

## Introduction

Sports fans enjoy watching games in various venues, such as their homes, sports bars, workplaces, and sports stadiums. Their viewing experiences vary widely depending on where they watch the game [1]. In particular, watching mediated (or televised) sports in public places (i.e., public viewing) provides a strong social experience, because viewers can share and affirm their dedication to the sport or team, as if they were spectating at the sports stadium [1–4].

This kind of public viewing has extended into online spaces on account of recent technological advances, such as high-speed Internet and Web 2.0 [5]. Sports fans can now watch sports games from any location using social TV services (e.g., Major League Baseball (MLB) TV and Naver Baseball) while interacting with other fans via online chat facilities. For example, fans can collectively root for their favorite teams from the workplace or while commuting. Social TV services provide a unique viewing experiences by giving viewers a similar degree of freedom as when viewing at home while offering the ability to share and affirm their devotion to a sport or team with other online fans.

Researchers have employed sports-related chat interaction data in various applications (e.g., to generate game highlights [6] or annotate games on video [7]). However, the factors affecting fan interactions during a sports games have not yet been explored. In general, fans react to games being viewed on-screen; thus, some aspects of games produce chat interactions. There are many variables relating to game content; therefore, our present goal is to identify how each variable affects these chat interactions. The results are expected to bring valuable insights to various application, such as by providing clues for real-time advertising or improving sports-related marketing [8]. Furthermore, existing applications using social response data—such as the above-mentioned highlights and annotations—can be improved by relating the interaction data analysis to the content information.

Based on the above considerations, we analyzed a large-scale chat dataset from Naver Baseball, the most popular website in Korea for watching live baseball games. We considered various factors (i.e., pre- and in-game factors) and built a multiple regression model that estimates the relationship between these factors and the volume of online chat messages. Pre-game factors are the statistics that can be measured before a game starts, such as team performance records. In-game factors relate to game content that can be measured during the game (e.g., score differences and changes in winning probabilities). We additionally performed regression analysis by employing different user groups based on chat activity and fan loyalty. The main findings of this paper can be summarized as follows:

- Our model shows that both pre- and in-game factors play an important role in explaining the volume of chat messages produced during game viewing. We found that the most influential factors affecting chat interactions are closely related to the performance records and chat interaction histories of teams, as well as to the dynamics of game events.
- The regression results with various user groups show low activity (groups who created only a small number of chat messages) or weak loyalty (groups who supported more than a few teams) are influenced more strongly by in-game factors. In contrast, pre-game factors are more important than in-game factors for groups with high activity or strong loyalty.

We believe that the above findings can be used in diverse application scenarios, including sports marketing and video annotations. To the best of our knowledge, this is the first large-scale study to attempt to identify key factors affecting chat interactions. Our large-scale data analysis complements the conventional approaches of small-scale user studies. Also, we present a language-independent analysis framework and provide the dataset we used for analysis [9]. We believe that such our efforts can contribute into extending existing knowledge about sports fans’ behaviors and sports analytics. However, because our study performed in a single site, the results need to be understood in its contexts and further studies are necessary for generalizability.

The remainder of this paper is organized as follows. We first describe related work and illustrate user interactions in Naver Baseball. We then present the candidate factors and build a multiple regression model from which we identify the key factors. Finally, we discuss the implications and limitations of our approach and present our conclusions.

## Background and Related Work

In this section, we review related work on chat interactions in TV and their applications. We then give an overview of sports analytics and illustrate their application to baseball.

### Chat Interactions in Sports Games

The usefulness of chat interactions on TV has been addressed by many researchers. Chat interactions can improve the viewing experience, because they help users to enjoy watching TV programs and understand the content [10]. Frequent chat interactions enable users to develop interpersonal relationships with other co-viewers [11]. To facilitate chat interactions on TV, researchers have proposed various social TV systems, such as CollaboraTV [12] and AmigoTV [13]. These systems provide various social features (e.g., status updates, community building, and communication) that encourage people to interact with other viewers [14]. In addition, chat interactions on TV have been analyzed from various standpoints, such as genres [15], interpersonal relationships [16], and communication channels [17]. Moreover, Geerts and Grooff proposed guidelines for increasing sociability in such social TV systems [14].

Earlier works have shown that sports are one of the most well-distinguished genres. First, people are more interactive while watching sports games [15]. Geerts et al. showed that communication patterns are mainly dependent on genres. In particular, the plot structure of the genre is an important factor in determining the viewers’ level of chat interactions while watching TV. Genres with an engaging plot structure, such as dramas or movies, permit fewer chances for synchronous chat interactions because they require more attention from the viewers. In contrast, genres with a short plot structure, such as quizzes and sports, provide more opportunities for synchronous interactions.

Second, people feel more comfortable talking with strangers while watching sports games [16]. In most genres, there are relatively few chat interactions among strangers, because people prefer interacting with family or close friends. However, sports fans enjoy watching games with strangers on account of the shared group identity associated with specific teams [18]. Further, in sports games, the number of co-viewers is more important for viewer satisfaction than the identities of the co-viewers.

Many researchers have focused on utilizing social interaction data in diverse application scenarios, such as supplementing TV viewing rates [19] and developing strategies for advertising [20]. The use of social commentary data from sports games has been reported in a number of earlier works. For example, viewer responses can be used to summarize TV programs [7, 21]. Additionally, various methods for extracting the most interesting moments from a video have been proposed [6, 22, 23]. Shirazi et al. evaluated a mobile app that facilitates social interaction in soccer games to provide an enjoyable viewing experience [24].

Our study advances this earlier work by analyzing a large-scale interaction dataset to build a regression model. From this model, the key factors affecting chat interaction data are identified. To the best of our knowledge, this is the first attempt at analyzing a large-scale dataset to identify the factors affecting chat interactions.

### Sports Analytics

Sports analytics is informally defined as the use of advanced quantitative methods to enable sports teams and their stakeholders to make better decisions [25], often using the massive data generated from sports games and operations. This use of sports analytics is not limited to the operation of game plays; rather, it includes the management and business operations of professional sports. In this more expansive definition, sports analytics includes not only quantitative models and statistical methods, but also data management, data visualization, and other information value chains that surround these models and methods [26].

Although studies applying quantitative models to professional sports first emerged more than 50 years ago, they received little public attention until the 2003 release of Michael Lewis’s seminal work, “Moneyball: The Art of Winning an Unfair Game.” This bestseller describes the story of how the Oakland Athletics baseball team used data and models [27]. Most MLB teams now utilize sports analytics as a normal part of their operations. Furthermore, more than half of National Basketball Association (NBA) teams use analytic tools on the playing side of their operations, whereas companies such as STATS LLC have installed cameras in NBA arenas and NFL stadiums to capture an increasing amount of in-field playing data [28, 29].

The growing demand for sports analytics is mainly due to the available data, which have increased exponentially over the past decade. With advances in information technologies, increased computing power, and reduced storage costs, the frequency and amount of information captured—as well as the stored data from all levels of sports operations—are far greater today than could have been imagined just a few years ago. The current challenge is how to effectively manage and extract meaningful information from this mass of data.

### Baseball Analytics

Owing to its discrete nature, baseball has been actively modeled in operations, applied statistics, and other mathematically oriented disciplines [30–34].

Bukiet et al. used a Markov model to determine the best batting order [35]. They evaluated the expected number of runs scored for each batting order, and showed that the difference between the best and worst batting orders amounts to only approximately 0.3 runs. They also suggested that the batter with the highest scoring index value, which is equivalent to the expected number of runs scored by an identical batter in an innings, might best be placed second, rather than fourth, in the lineup.

Hirotsu and Bickel [36] analyzed the impact of run limits, which is the maximum number of runs that can be scored in a half-innings in amateur baseball contexts. They proposed a unique Markov model for the ideal batting order by considering these run limits. Similarly, Robinson [37] focused on producing the optimal batting order in amateur youth baseball teams, whereas Cho et al. [38] and Moon et al. [39] applied Markov models to evaluate the batting orders of Korean professional baseball teams. Other Markovian models applied to baseball games are described in [40] and [41]. In our previous work, we attempted to accurately capture event-level influences by using existing Markovian models that mathematically describe the optimal batting order of a team and the winning rate.

## Chat Interactions in Naver Baseball

For our study, we employed Naver Baseball, a popular Korean website for watching live-streaming baseball games. The site has approximately 250,000 real-time viewers. In the following section, we give a brief overview of Korean professional baseball and describe how viewers interact with Naver Baseball interfaces.

### Korean Professional Baseball Overview

Professional baseball was established in Korea in 1982, and has become one of the most popular sports leagues in Korea. We analyzed regular-season baseball games from April 2011 to October 2012. In 2011 and 2012, there were eight teams in the league, and each team played 133 games in the regular season. There were typically four games per day, although no games took place on Mondays from April to October. After all games in the regular season were finished, the top four teams (based on win/loss records) qualified for the post-season. Our analysis covered all the regular games in the 2011 and 2012 seasons, but excluded special games such as all-star and post-season games.

The baseball league provides a useful environment for analyzing chat interactions in sports. A sufficient number of chat interactions during baseball games are readily accessible because there are 532 games per year; moreover, each game has a long runtime (3.5 h on average). In addition, baseball games are well-structured (e.g., a regular break at the end of an innings) and have many game events. Thus, we can systematically analyze chat interactions by considering various game-related factors.

### Naver Baseball: Live Streaming Baseball

Naver Baseball is a convenient online venue for the baseball viewing experience. It delivers live video streams from public broadcasting stations and supports multiple devices, such as PCs and mobile phones. With Naver Baseball, viewers can watch live baseball videos for free from any location, as long as they have an internet connection.

**Fig 1:**
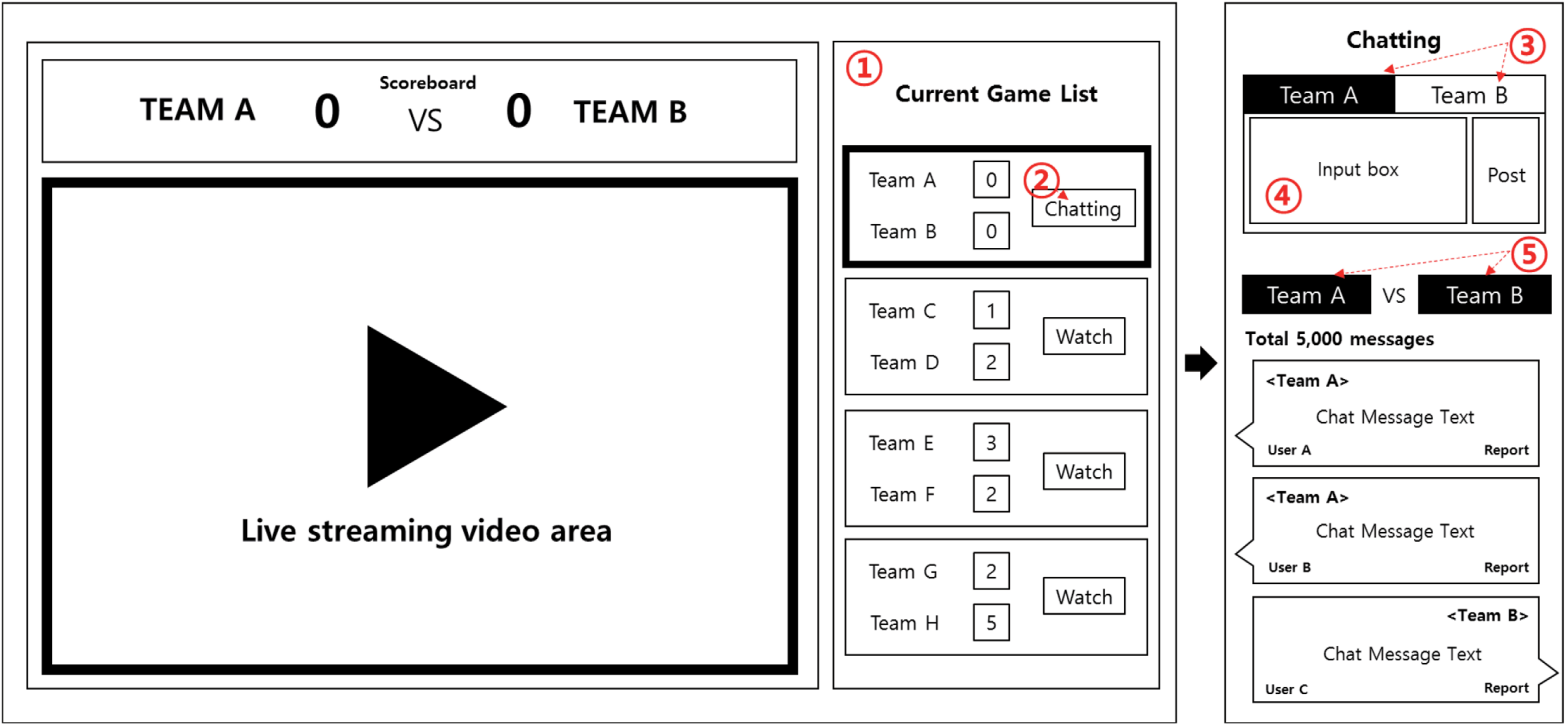
User interface of Naver Baseball. ①: Game list, ②: Chat button, ③: Favorite team selection button, ④: Chat input area, ⑤: Filter button to see chat messages on each team.

Naver Baseball supports real-time commentary features for live chatting. This function has been widely used in online mediated sports systems. Fig 1 shows the main user interface of Naver Baseball. When a user selects a baseball game, a pop-up window appears and streams the video of that game, as shown in Fig 1. The live video is shown in the left-hand panel of the pop-up window, and live score boards from the other games are displayed in the right-hand panel. Clicking the scoreboard of another game takes the user to the live streaming page for that game. If a user clicks the chat button, recent chat messages about that game are displayed. Because chat messages are placed in the right-hand panel, users can watch the live video while chatting online with other viewers.

To post a chat message, users are first required to sign-in the website. They are then asked to choose their favorite team, which is similar to choosing a home or away seat in a stadium (see ③ in Fig 1). Users can then write a chat message, which is displayed along with the selected team’s symbol. The maximum length of a chat message is 65 characters. To prevent spamming, users must wait 20 s before they can post another message. All recent chat messages are displayed by default. If users only want to chat with fans of a particular team, they can filter the chat messages by clicking the team’s icon.

### Data

To obtain the data for our analysis, we crawled live chat messages from Naver Baseball for two regular seasons (1,064 games from April 2011 to October 2012). The crawled data contained 510,945 unique users and 25,834,232 chat messages. Crawled chat messages consist of a game ID, user ID, chat message text, favorite team (i.e., the team selected when the user posts a chat message), and the time the message was posted.

In addition, we gathered textual game-related data to develop an in-depth understanding of the underlying game-related factors that influence the observed chat behavior. Korean Baseball Organization (KBO) provides a scoreboard and play-by-play data per game via a web site. The scoreboard data includes players’ records for each batting (e.g., hit, out, or homerun) and the scores that each team made. The play-by-play data provides more detailed information about each play such as play result per pitch (e.g., strike, ball or hit). We converted the original textual data to the standard numerical format of the Markovian model of analyzing the baseball [35]. Because the original texts are already semi-structured, we could easily convert the format. We would like to note that we did not perform any complex natural language analysis and our analysis method does not examine actual chat content in Korean. Converting game texts in a numeric format can be considered as a standard preprocessing method that are quite common in sports analytics. Other international baseball organizations such as MLB and NPB provide their game data in a similar format (e.g., Baseball-Reference.com [42])

Because our study analyzed user chat interaction data, it was necessary to address user privacy concerns. Thus, we collected only publicly accessible chat interaction data posted to Naver Sports (i.e., the 25,834,232 chat messages that we collected were open to the public). Furthermore, our dataset did not include specific demographic information about individuals. For privacy protection, the user IDs given by Naver Sports are encrypted. We used the encrypted user IDs to identify users, and received institutional review board exemption from the Korea Advanced Institute of Science and Technology (KAIST) for the Protection of Human Subjects to conduct this study. The dataset can be downloaded freely [9].

## Factors Affecting Chat Interactions

In this section, we explore the factors affecting online chat interactions. In our study, we first attempted to identify the factors that drive chat interactions. We considered diverse underlying factors, and conducted a regression analysis to examine the relationship between these factors and the volume of chat messages produced. Furthermore, we analyzed whether the factors had a different influence on user interactions based on the users’ chatting behaviors. We considered the users based on (1) how many games they chatted about (activity), and (2) how many teams they focused on in their chat messages (fan loyalty). A total of four user groups were selected: a head group (active users) *vs*. a tail group (less active users), and a loyal group *vs*. a non-loyal group. We analyzed the influence of the candidate factors on the chat interactions of each user group by conducting a series of regression analyses.

### Dependent Variable: Number of Chat Interactions

We compiled a list of dependent variables, specifically the number of chat interactions in a game. All variables were measured over a baseball game and normalized by the game time. Table 1 presents the statistics of the quantified chat interactions.

**Table 1:** Statistics of the Amount of Chat Interactions.

		**Median**	**Mean**	**SD**
All users	(510,946 users)	6281.03	6933.85	3525.18
**Activity**				
Head group	(8,380 users)	2980.52	3216.10	1519.47
Tail group	(452,385 users)	1033.59	1251.69	824.36
**Loyalty**				
Loyal group	(2,707 users)	1216.13	1311.97	623.18
Non-loyal group	(1,435 users)	302.75	335.74	176.39

*N* = 1,064 games

#### Chat Interactions of All Users

The first dependent variable was the mass interactivity of all users. For a given game, the interactivity was quantified by the number of chat messages per hour over the course of the game.

#### Chat Interactions of Special Users

**Fig 2:**
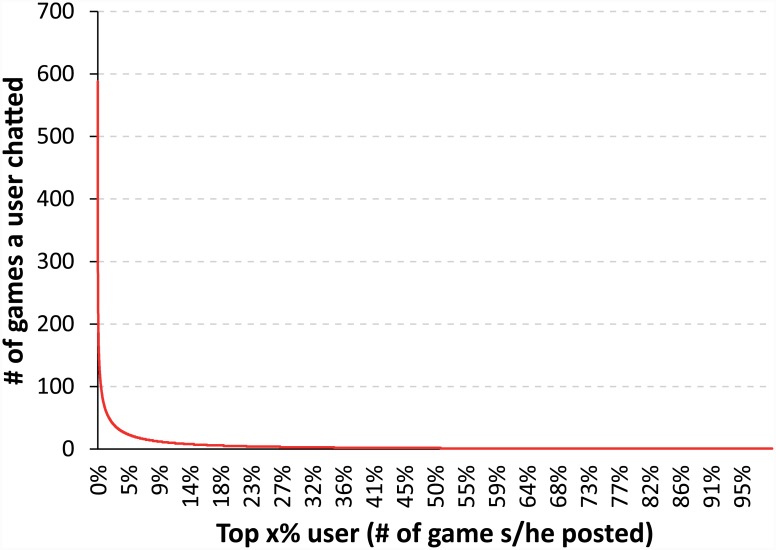
The User Distribution According to the Activity (# of Games a User Chatted).

The other dependent variables were the respective chat interactions of the four user groups introduced above. First, we selected the head group and tail group according to activity levels. A user’s activity was measured by how many games chatted about. Fig 2 shows the distribution of activity among users. We observed a heavy-tailed distribution, indicating that few active users chat during a large number of games, but most users rarely chat.

Based on this distribution, the head group (approximately the top 1% of users, >50 games) and tail group (approximately the bottom 90% of users, <10 games) were selected as follows:

- **Head group**: The head group included the active users who chatted in more than 50 games. Although this group contained relatively few users, they were responsible for a significant proportion of the chat messages.
- **Tail group**: The tail group included the less active users who chatted in fewer than ten games. A large proportion of users chatted for only a small number of games.

The number of chat interactions conducted by each group was quantified by the number of chat messages per hour over the course of a game.

Second, we selected a loyal group and a non-loyal group based on fan loyalty. We defined fan loyalty as how many teams a user typically selected for chatting. Naver Sports asks users to choose one team in a game via the team selection button (Fig 1). We assumed that loyal fans would tend to select their favorite team more frequently than other teams. A user’s fan loyalty was computed based on entropy. For a given user *u*, we calculated the fraction of chat messages related to team *t* by user *u* (denoted as *p*_*u*,*t*_). *p*_*u*,*t*_ was computed by dividing the number of *u*’s chat messages on team *t* (denoted as *C*_*u*,*t*_) by the sum of *C*_*u*,*t*_ over all teams. Using this team support distribution, we calculated the entropy of user *u*. The entropy was then normalized and negated to map the fan loyalty values to the range 0–1. The resulting equation is given as follows:

$$FanLoyalty_{n}=1-\left(\frac{-\sum_{t\in T_{u}}p_{u,t}\cdot \log_{2}p_{u,t}}{\log_{2}\left|T\right|}\right),\;p_{u,t}=\frac{C_{u,t}}{\sum_{t\in T_{u}}C_{u,t}}$$ (1)

where *T*_*u*_ is the set of teams chatted about by user *u*, and |*T*| represents the total number of teams (eight in our case).

Intuitively, the higher entropy, the greater the randomness. Therefore, entropy was maximized when the user uniformly supported all of the teams (with a probability of 1/8 in our case, because there were a total of eight professional baseball teams in Korea in 2011 and 2012). Fan loyalty ranged from 0 to 1. When a user chatted about a single team, the user’s fan loyalty was 1; a user who chatted about each team equally had fan loyalty of 0.

**Fig 3:**
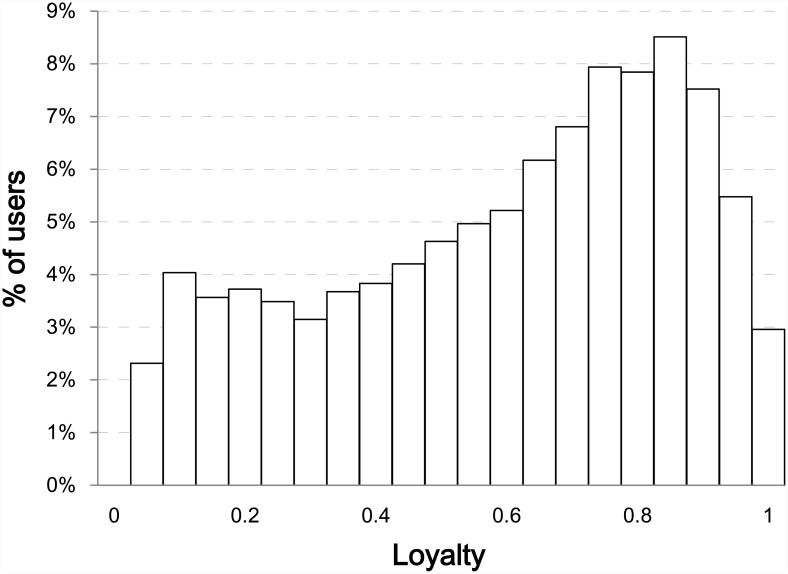
Fan-Loyalty Histogram with a Bin Size of 0.05 among the Users in Head Group.

Fig 3 shows the distribution of users in the head group according to fan loyalty. In this distribution, we only considered the active users (head group) to reliably calculate the entropy. Most users were very loyal, and 40.2% of users had loyalty scores above 0.75. However, there were a large number of users who chatted about various teams.

Based on fan loyalty, we divided the users in the head group into—a loyal group and a non-loyal group—as follows:

- **Loyal group**: The loyal group included users whose fan loyalty values were higher than 0.75. The users in this group concentrated on chatting about only one or two teams.
- **Non-loyal group**: The non-loyal group included users whose fan loyalty values were lower than 0.25. The users in this group chatted about three or more teams in approximately equal amounts.

Similar to the head and tail groups, the number of chat messages for each of these groups was measured by the number posted per hour during the course of a game.

### Independent Variables: Underlying Factors

The independent variables, i.e., the potential factors affecting chat interactions, were extracted from both the baseball game data and chat message data. The factors could be classified into two categories: pre-game factors and in-game factors. Pre-game factors are statistics that can be measured before a game has started, such as the team’s performance records. In-game factors relate to the game content, and can be measured during the course of the game, such as the score or number of game events. The basic statistics of the factors considered in this study are given in Table 2.

**Table 2:** Statistics of Underlying Factors.

	Median	Mean	SD
**Pre-game factors**			
*PF*_*recent*_*win*_*avg*	5.00	4.68	1.30
*PF*_*recent*_*win*_*diff*	2.00	1.88	1.38
*PF*_*season*_*rank*_*avg*	2.5	2.93	1.99
*PF*_*season*_*rank*_*diff*	1.00	1.74	2.05
*PF*_*team*_*popularity*	7340.03	7070.21	2058.31
*PF*_*match*_*popularity*	6972.19	7389.45	2687.43
**In-game factors**			
*IF*_*final*_*score*_*avg*	8.00	8.35	4.53
*IF*_*final*_*score*_*diff*	3.00	3.42	2.56
*IF*_*event*_*cnt*	81.00	82.60	9.76
*IF*_*event*_*impact*_*sum*	1.10	1.29	0.65

*N* = 1,064 games

#### Pre-Game Factors

First, we considered two factors related to the recent performance record. The recent performance of two teams in a game could impact users’ chat interactions (PF_recent_win_avg). For example, if both teams had won recent games, users might expect to see more enjoyable content during the match. Thus, the number of chat messages during that game may be enhanced. Additionally, differences in the mood of the players could be important (PF_recent_win_diff). Consequently, there might be less tension in a game if the recent performance of one team was significantly better than that of the other. These factors were computed as follows:

- **PF_recent_win_avg**: The average number of wins by each team over their past ten games.
- **PF_recent_win_diff**: The absolute difference in the number of wins by each team over their past ten games.

Second, we considered two factors relating to the win/loss ranking of the two teams in a game. Similar to the previous two factors, these factors likewise considered team performance. However, they additionally accounted for the overall records of the two teams prior to the current game. When the top-ranked teams played one another, users typically had high expectations of that game (PF_season_rank_avg). Such expectations might lead to more frequent chat interactions. Additionally, users may interact more frequently because of greater tensions when both teams had similar win/loss rankings (PF_season_rank_diff). We measured these factors as follows:

- **PF_season_rank_avg**: The average rank of the two teams in a game prior to the current game.
- **PF_season_rank_diff**: The absolute difference in ranks of the two teams in a game prior to the current game.

Finally, two factors relating to popularity were considered. We expected users chat more frequently about those teams who are more popular and drew more interactions in recent games (PF_team_popularity). In addition, the popularity of the match itself could influence the number of chat interactions (PF_match_popularity). A good example of this is the rivalry between certain teams, such as the New York Yankees and Boston Red Sox in MLB. These factors were quantified as follows:

- **PF_team_popularity**: The average number of chat messages related to each team in their past ten games.
- **PF_match_popularity**: The average number of chat messages posted during the past ten games between the two teams.

#### In-Game Factors

Unlike the pre-game factors, in-game factors are associated with the current game content. These factors are measured based on game content models that indicate how enjoyable a particular game is.

First, two in-game factors were computed at the level of the game score. We assumed that a higher aggregate score would result in more chat interactions (IF_final_score_avg) would be. Additionally, we expected that close games would lead to more chat interactions, because small changes in score could affect the outcome (IF_final_score_diff). These two factors were calculated as follows:

- **IF_final_score_avg**: The average of the final scores of the two teams in a game.
- **IF_final_score_diff**: The absolute difference between the final scores of the two teams in a game.

Second, two in-game factors were extracted at the level of the game event. These factors had higher granularity than the score-related factors. If the game contained more play events, it was considered to be more exciting and enjoyable. Thus, there would be more chat interactions (IF_event_cnt).

- **IF_event_cnt**: The number of batting-related events in a game.

Furthermore, many interesting events could occur, such as a team almost scoring. The existence of such events could impact the frequency of chat interactions.

- **IF_event_impact_sum**: To measure the impact, we adopted a fine-grained baseball model that evaluates the possibility of winning based on a Markovian model. This model returns the probability of winning after every batting event. We measured this factor by summing the increments in the winning probability on account of each event in a game.

Here, we briefly explain the Markovian model that evaluated the winning probability of an event. The Markov chain model was applied to imitate the progression of a half-innings in baseball, in which one team bats until there are three outs. The states of the Markov chain were defined to represent the positions of the runners on bases and the number of outs. In baseball, there are three bases that are either occupied by a runner or not. Therefore, there are eight possible runner states.

Hence, because there are three possible numbers of outs (none, one, or two), there are a total of 8 × 3 = 24 possible states for each innings. In addition to these 24 states, we added a state to represent the “three outs” as the 25th state. All the innings started with no runners and no outs, and ended with the three outs, with the 25th state being the absorbing state. The game advanced by transitioning from one state to another. Each transition had an associated probability. The transition matrix *P* matrix, consisted of 25 probabilities for all 25 states. Each transition probability was evaluated based on past data.

Note that the transition probabilities were assigned player-specific values, i.e., the probability that Player A would change the state from a one-out with first-base runner to a one-out with first- and second-base runners (in this case, the batter hit a single). This scenario is different from the identical state change of other players. Additionally, one transition represented the possible run scores resulting from the state change. If a state transition occurred, then there would be no outs with a third-base runner, representing a transition to the state of there being no outs with a runner. The latter state could only be reached when the batter hit a home run. With this transition, two scores were earned. Therefore, from each transition, we could estimate the runs scored.

**Fig 4:**
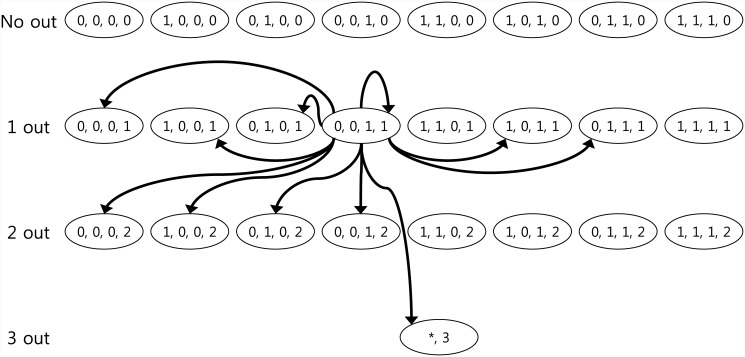
Markov Chain state space.

Fig 4 shows the Markov chain state space. Each state was written as (*x*_1_,*x*_2_,*x*_3_,*O*), where the first three elements (*x*_1_,*x*_2_,*x*_3_) refer to the base runner state and the fourth element is the number of outs. Specifically, *x*_1_,*x*_2_, and *x*_3_ represent the binary variables that indicate whether a base runner occupies first, second, or third base, respectively. For example, (1,1,0,2) corresponds to “runners on first and second bases, and two outs.” In addition, the three-out state, which is an absorbing state, is represented by (*,3).

Fig 4 illustrates an example of transitions from the state (0, 0, 1, 1) (“runner on third base, and one out”). The transition from (0, 0, 1, 1) to (0, 0, 0, 1) can occur when the batter hits a home run, or (very rarely) when the defense makes one or more errors, which enables both the runner and batter to score. This transition further indicates that the team scores two runs. Moreover, transitions to (1, 0, 0, 1), (0, 1, 0, 1), and (0, 0, 1, 1) mean that the batter has hit a single, double, or triple play, respectively, and the third-base runner came home, thus scoring one run. The other transitions can be easily explained with the run-scoring progression of the game.

With the player-specific matrix configuration, we could evaluate the expected number of runs scored for a specific lineup. We denoted the transition matrix *P*_*n*_ for each batter with a subscript *n* denoting the batter’s position in the batting order. We denoted the initial scoring matrix as *U*_0_, which consisted of 25 columns with *k* rows to represent the *k* − 1 number of runs (in our model, we set *k* = 21, because no game in recent years had more than 20 runs). We simply multiplied *U*_0_ by the transition matrix *P*1 for the first batter in the lineup, and then multiplied the result by transition matrix *P*2 for the second batter, and so on, until all nine batters had had the opportunity to bat. Play then returned to the first batter. We could thus evaluate the expected run scores from the corresponding probabilities [35]. With the matrix manipulation and information of a given condition, we could evaluate the probability of each team winning. A team wins a game by scoring more runs than the opposing team. Additionally, according to the tie-rule in Korean Professional Baseball, there could be a tie after the completion of the 12th innings. The detailed mathematical model and derivation of winning probabilities can be found in Jang and Yeo [43].

### Regression Model: Predicting the Number of Chat Messages in a Game

A series of multiple linear regressions were conducted to analyze the chat interactions of the five groups (i.e., all users, head group, tail group, loyal group, and non-loyal group). The regression models were constructed with various combinations of pre-game and in-game factors (PF+IF), pre-game factors only (PF_only), and in-game factors only (IF_only).

Our analysis identified several independent variables that were strongly correlated. In particular, the two highest correlations were observed between PF_match_popularity and PF_team_popularity, and between IF_event_cnt and IF_event_impact_sum. This is because one of these factors was a subset of the other. However, even these variables did not have a correlation that exceeded the 0.8 benchmark, which would indicate potential multicollinearity. In addition, the variance inflation factor (VIF) for each independent variable was lower than the benchmark of 10 for multicollinearity [44–47]. The highest value was 2.724. Therefore, our regression models were not affected by multicollinearity.

Table 3 lists the results of the multiple regression analysis. All models were significant, and the best model was obtained when pre-game and in-game factors were used. This model explained the intensity of chat interactions with games reasonably well.

**Table 3:** Multiple Regression Models with Chat Interactions of Different User Groups.

	All Users	Activity		Loyalty	
		**Head Group**	**Tail Group**	**Loyal Group**	**Non-Loyal Group**
**Goodness of Fit of the Model: *R*^2^**					
*PF* + *IF*	.601	.622	.481	.598	.405
*PF*_*only*	.495	.544	.358	.537	.304
*IF*_*only*	.108	.070	.133	.061	.095
**Standardized Beta Coefficient in the Model Using *PF* + *IF***					
*PF*_*recent*_*win*_*avg*	.140**	.221**	-.031	.175**	.155**
*PF*_*recent*_*win*_*diff*	-.028	-.028	-.026	-.063*	.050
*PF*_*season*_*rank*_*avg*	-.112**	-.144**	-.047	-.087*	-.119**
*PF*_*season*_*rank*_*diff*	.046	.015	.084*	-.014	-.008
*PF*_*team*_*popularity*	.320**	.327**	.220**	.382**	.188**
*PF*_*match*_*popularity*	.440**	.451**	.420**	.418**	.391**
*IF*_*final*_*score*_*avg*	.026	-.004	.073	-.005	-.014
*IF*_*final*_*score*_*diff*	-.028	-.031	-.025	-.041	.002
*IF*_*event*_*cnt*	.057	.086**	.012	.076	.062
*IF*_*event*_*impact*_*sum*	.264**	.209**	.304**	.181**	.283**

*N* = 1,064,

*: p-value <.01,

**: p-value <.001

## Discussion

### Dominance of Pre-Game Factors

We found that pre-game factors had a stronger influence than in-game factors. The fraction of variation in chat interactions was greater when only pre-game factors were used (PF_only) than when only in-game factors were used (IF_only).

This pre-game factors dominance was also observed when the beta coefficient values were evident in the model. Unlike the in-game factors, most of the pre-game factors were strongly correlated with the number of chat messages posted by all users.

The pre-game factors relating to popularity showed a strong correlation with the number of chat interactions during a game. In particular, the pairing of competing teams (PF_match_popularity) played a critical role in determining the volume of chat messages. Similarly, the popularity of the two teams in a game (PF_team_popularity) was a good predictor. When more popular teams played one another, the chat interactions were more frequent.

The number of chat messages was also significantly correlated with the performance of the two teams in a game. The chat interactions were more intensive when two teams had won a number of their recent games (PF_recent_win_avg). Additionally, the win/loss rankings of both teams were negatively correlated with the number of chat interactions, indicating that the closer the team was to the top of the rankings (e.g., first- or second-ranked team), the more interactive the viewers were (PF_season_rank_avg). Viewers expect higher quality plays and greater enjoyment when the two teams involved have exhibited a higher level of performance. Such expectations encouraged viewers to chat more about that game.

Among the in-game factors, IF_event_impact_sum was a significant predictor. This indicated the necessity of a fine-grained game model to properly account for chat interactions. When a game contained a number of interesting events that contributed to the outcome, a large number of chat interactions were observed. However, the other in-game factors, such as counting scores or play events, showed no significant correlations.

### Head Group with Pre-Game Factor (Game Expectations) and Tail Group with In-Game Factor (Game Content Quality)

Like the chat interactions of all users, the best model for the head and tail groups was built using both pre-game and in-game factors. The dominance of pre-game factors over in-game factors was again evident.

We found differences between the head group and tail group. Interestingly, the pre-game factors (expectations of the game) were more influential in the head group, whereas the in-game factors (game content quality) were more influential in the tail group. The PF_only model accounted for a larger proportion of chat interactions in the head group than in the tail group. On the other hand, the IF_only model fitted the chat interactions of the tail group better than those of the head group.

The beta coefficient values also showed that pre-game factors and in-game factors were comparatively well associated with the head group’s interactions and tail group’s interactions, respectively.

Team performance was a stronger predictor of chat interactions in the head group than in the tail group. PF_recent_win_avg and PF_season_rank_avg were strongly correlated with the chat interactions of the head group. This indicates that active users interacted more when the two teams were better ranked and had recently won many games. The tail group’s chat interactions were significantly associated with only the PF_season_rank_diff. Less active users were more interactive when the gap between the teams’ rankings was wider. However, this result also showed that the game content (i.e., in-game factor) was influential in the tail group. We analyzed the tail group’s chat messages to examine why PF_season_rank_diff was related to the tail groups’ interactions. As a result, we found that the gap between the two teams’ rankings contributed to the dramatic moments of the game. The tail group chatted more frequently when a team beat a higher-ranked team. PF_season_rank_diff was eventually related to the game results (i.e., game content), even though it was one of the pre-game factors.

In addition, the pre-game popularity factors were more influential for the head group. PF_team_popularity and PF_match_popularity showed strong correlations with both groups. However, their beta values suggest that they were more influential for the head group.

In contrast, in-game factors were more closely associated with the tail group’s interactions. The IF_event_impact_sum was strongly correlated with both groups. However, its correlation value for the tail group was higher than that for the head group. In addition, IF_event_cnt was significantly related to the head group’s correlation values.

Overall, the results with in-game factors indicate that consideration of the number of factors for conversation (i.e., the number of events) is essential for active users’ interactions, whereas events connected to the game result are influential for less active users’ interactions.

Such differences between the head and tail groups can be explained by their chatting tendencies during a game. The users in the head group were very active, so they chatted throughout the game rather than only at specific moments. Thus, it was more important for the head group to select interesting games to chat about, rather than to select dramatic moments within those games. However, the tail group users were different. Because they were less active, they needed sufficiently exciting game content to prompt their interactions.

### Loyal Group with Pre-Game Factors (Game Expectations) and Non-Loyal Group with In-Game Factors (Game Content Quality)

Generally, both the loyal and non-loyal groups showed similar results because they were selected from the head group. However, we also found significant differences between the two groups. In general, pre-game factors motivated the loyal group to be more interactive, whereas the in-game factors related more to the non-loyal group’s interactivity. PF_only showed that the total variance of the loyal group’s interactivity could be better explained by pre-game factors than that of the non-loyal group. On the other hand, the IF_only model accounted for the non-loyal group’s interactivity better than that of the loyal group.

The beta coefficient values also illustrated the different influence of the pre-game and in-game factors between the two groups. First of all, the popularity-related factors were more influential in the loyal group than the non-loyal group. In particular, the non-loyal group was less closely associated with PF_team_popularity. This indicates that loyal fans considered the recent popularity of their favorite team and matches more seriously.

Additionally, the performance-related factors indicated the differences between the groups. The loyal group was sensitive to the team’s recent record, whereas the non-loyal group considered the win/loss rankings. In other words, the recent performance of their favorite team was more crucial for loyal fans than its season rank. This may reflect the different motivations of the loyal group and non-loyal group. The loyal group’s main interest is to see their team win a game, rather than the team’s overall ranking. Therefore, they became more active when their team had been winning recent games.

Among the in-game factors, IF_event_impact_sum was the only significant predictor for both groups’ interactivity levels; however, its influence was different for each group. The influence of IF_event_impact_sum was much stronger in the non-loyal group than in the loyal group. The non-loyal group’s interactivity changed significantly with the dynamics of the game.

### Generalizability

As with single-site work, the generalizability of this work is limited; thus, additional research on other online sports-viewing sites—such as YouTube Live and MLBtv—is necessary. It would be especially interesting to analyze different types of chat interactions in various online sports-viewing services. For example, Naver Sports and YouTube Live support chat interactions among a large number of co-viewers by providing live streaming videos, whereas second-screen social TV services, such as Twitter and GetGlue, provide public spaces for chat interactions among TV viewers. In addition, cultural factors could have affected our study results. Even though the psychology of sports fans is generally considered to be cross-cultural, there could be some unique aspects of Korean baseball games (e.g., the tie-rule, regional tensions) that biased the chat interactions. Therefore, it is necessary to perform comparative studies of multi-cultural datasets.

However, we believe that our study and dataset can be helpful for international research communities, by considering prior studies on social media and information systems in specific languages and nations. First, researchers have been using national social media to design and evaluate new algorithms and systems (e.g., Sina Weibo in China and Kakao Story in Korea). For example, Zhao et al. [48] analyzed Chinese tweets in Weibo to build a real-time sentiment monitoring system. Park et al. [49] used A Korean Facebook users’ dataset to identify depressive symptom—related features. Researchers showed that local social media can sometimes provide useful indicators for detecting offline events; e.g., analyzing Japanese tweets to detect earthquakes [50] and inferring air quality based on Chinese tweets in Sina Weibo [51]. Although non-English languages were used for system design and evaluation, we can directly use the results when designing English language based systems. We believe that our models can be applicable for analyzing other language based systems. Furthermore, our dataset will be valuable to other researchers, since they can investigate interaction statistics (e.g., chat frequency) and time-series data for various purposes (e.g., automatically extracting highlight moments), even though chat content cannot be examined. Note that the WISE international conference holds Challenge Events [52] every year, and non-English datasets are often offered for data mining challenges.

Second, researchers have been investigating socio-cultural usage patterns associated information technologies, which is often of great interest to the Human-Computer Interaction (HCI) researchers and social scientists. Qu et al. [53] analyzed Sina-Weibo to identify how Chinese netizens used microblogging in response to a major disaster of the 2010 Yushu Earthquake (e.g., by studying message types, topical trends, and information dynamics). Nam et al. [54] analyzed the characteristics of user behavior (e.g., knowledge generation and user participation) in Naver Knowledge-iN, the largest online Q&A community in South Korea. Researchers studied Weibo datasets to analyze generic information diffusion as well as sentiment diffusion over social media [55, 56]. We believe that other researchers can leverage our large-scale, longitudinal dataset to investigate various socio-cultural usage patterns of social TV systems, which have been rarely explored due to lack of public datasets.

Third, we believe that our work provides an useful research framework for further studies to extend generaizability. Our analysis framework measures three variables (e.g., pre-game factors, in-game factors, and interactivity) and then performs a series of linear regression analysis to clarify correlations between the factors and the interactivity. We note that our framework is independent on language, and this can be helpful in conducting following studies in other countries. The variables we used for analysis covers quantitative aspects on the game and chatting data. For example, we used our dependent variables (e.g., interactivity), PF_team_popularity, and PF_match_popularity by counting the number of chat messages. Also, the dataset used to evaluate the winning probabilities of the baseball are certainly of interest to the international research communities. The dataset was organized in the standard format of the Markovian model of analyzing the baseball which was first proposed by Bukiet [35]. Since the rules and regulations of the baseballs of Korean Baseball League in South Korea are quite similar to the those of Major League Baseball in the US and Nippon Professional Baseball in Japan, the dataset can be used for any baseball related research regardless of the leagues.

## Limitations and Future Work

As mentioned earlier, our study results need to be understood in the context of Naver Sports and further studies are necessary for the generalizability. Furthermore, we will perform content analysis on chat interactions during sports games. Our current study did not adopt complex natural language processing techniques. However, sports-related chat messages contain expressions that are unique to sports viewing. Analyzing the chat expressions that occur frequently under various contexts via computational approaches (e.g., automatic classification of content types or sentiments) would enable new application services.

We also believe that enhanced understanding fostered through further research can extend existing sports video applications based predominantly on chat message volume (e.g., detecting events [6, 57] and summarizing sports games in various aspects [21]). For example, we found that the tail group was more sensitive to the game event (i.e., in-game factors) compared with the head group; therefore, the tail group users likely contributed more to detecting important events in the game. Analyzing different types of sports-related chat interactions can provide other useful insights for improving existing sports video applications. We believe that the direction of our research will not only help advance sports marketing and social television analytics, but also provide valuable insights into designing user interfaces for sports games for social TV, thereby enabling user interaction methods that facilitate chat interactions.

## Conclusion

Technological changes and cultural practices largely shape the global sports media landscape. In particular, the advent of social media and social TVs (e.g., Twitter and Naver Sports) have significantly increased the ability of sports fans to publically express their identity and feeling and to collaborate with other fans [58]. Thus, researchers have been very interested in sports fans’ online behaviors [12, 13, 15, 16], and multiple related studies have been conducted in different cultures [59, 60]. In our work, we identified the key factors (i.e., pre-game and in-game factors) that affect online fan interactions over social TV. For this end, we analyzed a large-scale, longitudinal dataset from Naver Sports and built a regression model that predicts the amount of chat messages in a game. We explored various pre-game and in-game factors and leveraged Baseball analytics (e.g., a Markovian model that evaluate the winning probability of an event).

In sports analytics, researchers have made significant efforts to model diverse sports games based on game statistics; however, online chat interactions have rarely been considered. Chat interaction data have mostly been studied in sports marketing analytics (e.g., analyzing the effectiveness of advertisements during sports games and analyzing the sentiments of sports fans) and social TV analytics (e.g., annotating or highlighting videos using social response data). Our work has attempted to bridge the gap between sports analytics and other fields by applying sports analytics models to identify the key factors influencing chat interactions. This work can be considered an initial step toward exploring the detailed relationships between game factors and chat interactions.
