# Correction: What Makes Sports Fans Interactive? Identifying Factors Affecting Chat Interactions in Online Sports Viewing

**DOI:** 10.1371/journal.pone.0154550

**Published:** 2016-04-25

**Authors:** The PLOS ONE Staff

There are errors in the Funding section. The correct funding information is as follows:

This work was partly supported by Institute for Information & Communications Technology Promotion (IITP) grant funded by the Korea government (MSIP) (No.10041313, UX-oriented Mobile SW Platform) and the Sports Science Convergence Technology Development Program of the National Research Foundation of Korea (NRF) funded by the Ministry of Science, ICT & Future Planning (NRF-2014M3C1B1034033).

The publisher apologizes for the error.
